# Medical Miscellanies

**Published:** 1817-06

**Authors:** 


### PART IV.

### MEDICAL MISCELLANIES.

Quicquid agunt Homines.

### *Some Observations on Chorea; and on the Connection between the Vascular and Nervous System.* By Thomas Seeds, M. D.

It is certainly a most auspicious circumstance for the improvement of medicine, that the value of anatomy is now beginning to be more fully appreciated, and that a general persuasion prevails, that every action of living sentient bodies is more or less under the influence of those powers which alone distinguish the animal from the vegetable and mineral.

Though such men as Cullen and Brown could not have been ignorant of these plain and obvious truths, yet, no impartial man will affirm, that their far-famed systems were framed in just subserviency to them. Indeed, the attempt to systematise medicine, as it now stands, is as absurd as it would have been to have done so by chemistry, in the sixteenth century; or electricity at the beginning of the seventeenth. Can it be a matter of surprise, that the true knowledge of diseases is so imperfect, when numbers of medical men, and those of no mean celebrity, have, in all ages, been grossly ignorant of the composition of the human machine; and even those who knew something more of it, never suffered this knowledge to guide, restrain, and direct their enquiries and speculations, thus verifying the sarcasm of the French wit, who affirmed, that physicians were men who poured drugs, the nature of which they were ignorant of, into a body, the composition of which they were unacquainted with.

It appears to me, that nothing can raise medicine from this painful and degrading state of uncertainty, but a minute and careful attention to the structure and functions of the nervous and vascular systems, and a careful examination of their connections and reciprocal influences on each other. This would furnish us with Ariadne's clue; by the help of which, we might hope to pass the labyrinth, whose windings, at present, amaze and perplex us.

It has been well said, that "to doubt is the beginning of wisdom;" and no where is rational scepticism more requisite than in medical investigations. Authority should, I conceive, be held absolutely as nothing, unless supported by plain, well-established facts and principles.

The man who establishes, on sure and solid grounds, one principle of great and universal applicability, is more a benefactor to the medical profession, and to the world at large, than he who offers to notice five hundred new remedies.

The nervous system, (by which is meant the brain, spinal marrow, and nerves proceeding from both) exerts a more or less direct influence on every action of living sentient beings. The nervous and vascular systems mutually and reciprocally affect each other, and that with great rapidity and uniformity. Every part of the nervous system is under the influence of the vascular, inasmuch as the pia mater covers not only the brain and medulla spinalis, but likewise their nerves. The nerves maintain a constant intercourse between different parts of the body and the nervous centre.

Increased arterial impulse on the nervous system induces increased action. Venous turgescence tends to produce apoplexy and insensibility. On a proper equilibrium between the vascular and nervous systems health depends, and *vice versa*.

When an organ is disordered in function and not in structure, we must look to the state of the moving powers to discover the cause; disorders of function precede morbid organic changes.

The encephalon furnishes nerves to the organs of sense; the muscles of the face and head; and in part, those to the viscera. The medulla spinalis gives origin to the nerves of the trunk and extremities, and, in great part, to the internal organs; and is, by means of the grand sympathetic, connected with every internal organ, without exception.

By the origin of a nerve is meant, its visible offset, or place at which it leaves the brain or spinal marrow. The influence of the nerves on the brain and spinal cord, and of them on the nerves, is mutual and reciprocal.

To describe the phænomena of a disease so generally and extensively known as Chorea, appears superfluous; a few remarks may, however, be made on some leading circumstances connected with it. The period at which its attacks supervene, varies usually from the eighth to the fourteenth year; though sometimes it is said to come on a little later, especially in females. Sleep generally procures a truce to the agitations and uneasy sensations in the feet and limbs. A sensation as it were of air passing up the spine, various disorders of the digestive organs, and several irregular nervous symptoms, in different cases, precede the attacks of chorea. In process of time, if the violence of the disease be not previously checked, well marked epilepsy, fatuity, or a certain degree of emaciation of the body, may supervene. At puberty, this disease usually gives way; and it is alleged, that should it have attacked an adult, a considerable change in the habits of life materially assists in the cure. Danger from this disease, *simpliciter dictum*, is rarely to be apprehended; though, in course of time, a transition into dangerous or fatal disorders may take place. An absence of those signs, denoting great oppression of the head, regularity of the digestive functions, and the continuance of the plumpness of the body and the firmness of its muscles, are favourable and auspicious circumstances; *et e contrario*, epilepsy, great head-ache, and very violent spasms of the muscles of the face, are all decidedly unfavourable signs. I think it will be admitted, that the contraction of any muscle or muscles is dependent on the nerve or nerves supplying them; and that if these nerves are excited or irritated, correspondent actions will take place in the muscles to which they are distributed. Now we know of no circumstances capable of thus influencing the nerves, excepting their blood-vessels; we know likewise, that, in many cases, arterial impulse gives rise to increased action; but, above all, dissection has amply shewn and clearly demonstrated, that wherever any organs have been spasmodically affected, a turgid state of vessels at the root of the nerves, and not unfrequently an effusion of water, will there be found.

This state I have myself frequently seen in animals (both human and canine), whose death had been preceded by convulsions. My friend, Dr. Moulson of Chester, has witnessed such dissections, conducted by Dr. Saunders; and more than that, he induced convulsions in a healthy horse and in some dogs; and on dissection found, that the origins of the nerves of the convulsed organs were affected just as those of the patients whose bodies he had previously examined. Numerous observations on this point are contained in the works of Galen, Alexander Trallianus, Morgagni, Lieutaud, and others, which, for fear of trespassing too long on the patience of the reader, I shall forbear to cite. Suffice it to say, that their observations are quoted, at length, in Dr. Moulson's thesis on chorea. From a due consideration of these facts and observations, I am decidedly convinced, that the source of all spasmodic movements is to be found in the state of the origins of the nerves, and that it is as absurd to look elsewhere for them, as it would be for a surgeon, who had a patient with a dislocated arm, to look to the thigh to discover the nature of the accident.

Various irritations, as of worms, dentition, sordes in the bowels, fear, and other emotions of the mind, are all capable of inducing the irregular movements constituting chorea; these, I take it, all operate in one and the same way, *by disturbing the balance of the circulation, and inducing irregular distributions of blood.* With regard to dentition, Hippocrates affirms, that the passage of the canine teeth through the gums produces the greatest degree of irritation; I know not if it be so; certainly it is an object of popular belief. It does not appear foreign to the subject here to remark, that now and then, on cutting the dentes sapientiæ, great irritation supervenes, and a long train of the worst evils, marking the progress of dentition in the young. One well marked instance of this I have heard of in a young lady of good family and fortune in the West of Scotland.

As for the cure of this complaint, I conceive that our object should be threefold; first, to remove or obviate any existing irritation, which may be supposed to produce or maintain the disease; secondly, to relieve the origins of the nerves affected; and, finally, to equalize the circulation, and thus by procuring and supporting general action, to relieve local turgescence and prevent their occurrence.

As for the means of accomplishing the first intention, they are too obvious to need much comment; suffice it to say, that if the period of the attack favour the idea of dentition exciting the complaint, the gums should be scarified, &c. Should the bowels be loaded with sordes, or irritated by worms, means should be adopted for removing and obviating these; ever bearing in mind, that in such cases, the only legitimate object we have in view, in such purgations, is to establish the ordinary and healthy action of the intestines, to the attainment of which, applications to the origins of the nerves frequently contribute.

As for the last object in view, which I hold to be by far the most important: Leeches or cupping glasses should be applied as near as possible to the offsets of the nerves, whose functions are deranged. This should be followed up by powerfully stimulating frictions; or, what is perhaps better, a blister of greater or less length, according to the extent of the disease. Should symptoms evince considerable cerebral affection, the timely abstraction of blood topically with other external applications are strongly indicated. The remedies necessary for producing a general equal flow of blood throughout the system must vary with the particular case: sometimes the cold bath, under, however, considerable restrictions and modifications; tonics, as the oxyde of zinc; antispasmodics, as the warm bath, opium, hyoscyamus, and a multitude of other remedies, *quæ nunc repetere longum est*. Much has been said on the efficacy of purgative medicines. It may not be amiss however to observe, that calomel, either alone or in conjunction with other cathartics, has been usually administered by the advocates for extreme purgation. Now it is well known that all mercurials, in a greater or less degree, excite fever or increased but general action; and I feel strongly disposed to think, that thus, by an unlooked for change, a disease has been removed which the mere fulfilment of the physician's intention would never have effected. No less a man than Hippocrates has remarked, that if convulsions succeed fever, they are fatal; while on the other hand, fever supervening on convulsions or tetanus, removes the previous disease. *Sect.* 4. *Aph.* 47 *and* 49.

When my friend Dr. Saunders's great and long expected work comes out, these, and a multitude of similar topics will be clearly illustrated, ably discussed, and I may say, satisfactorily demonstrated. The appearance of this work will, I confidently anticipate, form an epoch in medicine, and the world at large will perhaps rival me in admiration of his great talents and unwearied labours.

THOMAS SEEDS.

London, May 1, 1817.

### To the Editors of the Medico-Chirurgical Journal & Review.

GENTLEMEN,

I have just read, in your Journal of the present month, the proceedings had on the trial of Mr. Donnall for the alleged "wilful murder of his mother-in-law, on the 3d of November last, by administering arsenic in a cup of cocoa, upon bread and butter, or in some undiscovered manner;" and, in my humble opinion, there never was a more complete exculpation from guilt than that which was afforded to the accused by these proceedings.

Notwithstanding the satisfactory circumstance of his acquittal, much regret (I might say horror) naturally suggests itself, when we contemplate the life and character of an innocent individual as being dependent in so great a degree upon the shallow testimony of perhaps deficient judgment, or upon the vague deductions of superficial and ungrounded investigation.

It appears that Dr. Edwards, in his evidence before the court, stated, "he had not attempted to reproduce the arsenic (the only infallible test he could have instituted), not having a sufficient quantity." Admitting that to have been the case (for the *fact* is tenable on admission only), so great a chemist as Dr. Edwards would appear to be, should have known, as well as Dr. Neale (and he might have known it by a reference to almost any author that has written on the subject of poisons), that the tests he adopted are never implicitly confided in; but that, on the contrary, they are generally liable to distrust, inasmuch as it has been clearly demonstrated, that similar results will be obtained from them where it is obvious no arsenic could be present. Dr. Edwards should have known also, that the precipitates obtained from a solution of arsenic by the means he employed, will, when exposed to a sufficient degree of heat, sublime and *emit "the garlic smell."** Although this circumstance would not have been received as conclusive evidence, on which to have at once pronounced the detection of arsenic, still however it would have been deemed a valuable auxiliary in support of his pre-entertained opinion, and would moreover have evinced a laudable desire, on the part of Dr. Edwards, to give the subject all the attention that its awful importance demanded.

On the bare authority of *his* never having seen death produced by cholera morbus, "in a shorter time than three or four days," Dr. Edwards presumed to give the opinion, "that that disorder was not the cause of the death of Mrs. Downing," she having died within that period!" Now, without the least wish of objecting to the validity of Dr. Edwards's *general* statement, I take the

---

* Vide Edinburgh Medical and Physical Dictionary, under the head Arsenic.

liberty of suggesting if it would not, in this *particular* instance, have been quite as consistent with propriety and the weightiness of the subject under investigation, had Dr. Edwards corroborated that opinion by the testimony of some well accredited author, as it was to suffer it to hinge on his own evidence alone? Dr. Edwards, however, did not do so; and indeed the testimony of authors, as well as the testimonies of the enlightened physicians on the opposite side, are against him.

The testimony of authors is, that when cholera morbus terminates fatally, that event not unfrequently happens *within twenty-four hours.* Probably much stress is laid, by persons unacquainted with disease, on the circumstance of Mrs. Downing having been similarly indisposed about a fortnight before the fatal attack, and that too, after having *supped* at Mr. Donnall's house. But there is nothing in this seeming coincidence that can, in the least degree, attach criminality to any one; indeed, it goes very far (as I shall presently shew) in exculpation of the accused.

It must be recollected, that there are two species of cholera described—" 1. Cholera spontanea, which happens in hot seasons without any manifest cause. 2. Cholera accidentalis, which occurs after the use of food that *digests slowly*, and irritates the primæ viæ.

A modern author, of much and deserved celebrity, (Dr. Thomas) further observes:—" There are some people who are subject to *periodical* attacks of cholera, returning by intervals of a *few weeks*, producing, for two or three days, sickness and vomiting; increased heat of skin and quickness of the pulse; white tongue, and thirst."

These facts then, deliberately considered, with reference to Mrs. Downing's first illness, and her subsequent attack, will leave but little doubt on the minds of thinking persons, that her former indisposition was occasioned (and her having eaten supper strongly favours the conclusion) by the use of food that did not easily digest; and that a recurrence of the malady was produced by a similar exciting cause, (for it is in evidence that she had eaten *heartily* at dinner of " *rabbits smothered with onions*") at a period too, when the disease would, from its peculiar character, be likely to return.

The symptoms of Mrs. Downing's final seizure, as described by Mr. Donnall, were indubitably such as constitute cholera morbus, and the treatment he adopted was not incompatible with the usual mode of treating that complaint; yet Dr. Edwards has not, in any part of his evidence that I can perceive, attempted to controvert the statement of Mr. Donnall, by shewing, that dissimilar symptoms, indicative of other disorders than that which Mr. Donnall had suggested, came under his observation—or that any circumstances arose at the time to impress his mind with a suspicion of arsenic having been administered. Another consideration, of infinite importance in this momentous affair, is, that

Dr. Edwards has omitted to state what were the *external* appearances of the body after death. No circumstance, however trifling, that would, in any view, tend to elucidate an apparent mystery, should have been withheld. I have thus cursorily brought before your readers a subject of the highest importance to the interests of the community at large, as well as to individual members of it, in the hope that it will henceforth be discussed and settled in a manner at once satisfactory and beneficial to all parties.

I am, Gentlemen,
Your very obedient Servant,
W. NORMAN.

Langport, 13th May, 1817.

---

We have received the following paper from Glasgow, with a request to have it inserted in our Journal. We readily concur in any measure which will give publicity to the candid and forcible statement of the just claims of so respectable a body as the University of Glasgow.

*Memorial and Representation of the University of Glasgow, against a Bill presented to the House of Commons, during last Session of Parliament, respecting the Practice of Surgery.*

THE Memorialists most respectfully beg leave to represent that the clause of the Bill in question, which proposes to limit the power of granting Diplomas in Surgery to the Royal Colleges of Surgeons in London, Edinburgh, and Dublin, will, if passed into a law, be a direct and alarming violation of the charter and rights of their Corporation; which, by every tie of duty and honour, they deem themselves bound, as far as lies in their power, to guard and maintain.

Their foundation charter, granted in the year 1450 by the Pope, according to the custom at that time, and at the desire, as the charter bears, of James the IId. of Scotland, and expressly modelled on that of the more ancient and celebrated University of Bologna, was ratified by that monarch; and having been recognized and confirmed by his successors, and by acts of parliament, and acted on, as a valid and undoubted deed, in all the subsequent and even recent transactions between Government and the University, has become a part of the law of the land.

By that charter, there is conveyed to the University, *inter alia*, the unreserved and unqualified privilege of teaching, of examining, and of granting *brevia* as they were then called, or *licences* as in the charter they are explained to be, *in quavis licita Facultate.* And these brevia authorised the persons who were found qualified to hold them, to exercise their respective Faculty or Art within the bounds of the Apostolic See, which, at that time, included Great Britain and Ireland.

In exercising this privilege, the University has been guided uniformly by one rule: *viz.* Not to grant a brevium in any branch of science or art, till an establishment was made for the regular teaching of the same, in the University. It appears from the records, which, if required, will be produced, that for a long time after the University was instituted, the only degrees conferred were those of Bachelor and Master of Arts, and Doctor in Divinity; because, at that time, the learned languages, Philosophy and Divinity, were the only branches taught in the University. It was not till the reign of George the First, that the University obtained the foundation of a Professor of Laws; after which, the degree of Doctor of Laws seems to have been occasionally conferred: at length, in the year 1716, a Professorship of Medicine was established; and in the year 1720, a Professorship of Anatomy and Botany was added. The University then began to confer degrees in Medicine: since that time, establishments for the other branches in the department of Medicine have been added, and the conferring of medical degrees has been regular and frequent.

The Universities on the Continent, especially in Italy, have been in the habit of granting brevia or diplomas in surgery. This University has not granted any; not because they entertained any doubts with regard to their right, but because they had not a distinct establishment for surgery:—Their procedure in this instance will illustrate their general practice. Their students were taught surgery by their Professors of Anatomy, along with anatomy; and the present Professor of Anatomy gave lectures and demonstrations on surgery for several years, at a separate hour, and as a separate course: yet, as there was no Professor of Surgery, the University did not think themselves warranted in deviating from their general custom.—About a year and half ago, however, his Majesty's present Ministers, taking into consideration the importance of surgery, recommended and obtained from the Crown the appointment and endowment of a Professorship of Surgery in the University; and the Medical Professors, persuaded that the University no longer could, consistently with their oath, *de fideli*, decline granting diplomas in surgery, were preparing to bring the matter before the Senate of the University, when they learned that a bill was brought into parliament, to regulate the practice of surgery, which caused them to delay proceeding farther in the business, till the will of parliament should be known.

The Memorialists have thus candidly stated the aukward situation in which they find themselves placed. They have, to the best of their judgment, conscientiously done their duty, in not granting brevia or diplomas, in any Faculty, till they had got provision made for the regular teaching of the same; and now, after they have got ample provision made for the teaching and examining in surgery, by the appointment of a separate Professorship, while the Professor of Anatomy is to continue to give his lectures and demonstrations in surgery also, they find, that by waiting respectfully for the determination of parliament, they run the risk of having their privilege of granting diplomas in surgery questioned, merely on the ground that they have not exercised it. If, however, the principles of the law of prescription be applied to matters of this nature, to which the Memorialists are persuaded they never were intended to refer, the consequences will be very serious, not only to this University, but to Corporate Bodies in general: for it is believed that no University or Corporate Body ever thought of exercising wantonly, or till occasion called, all and every right, and privilege, and power, with which, by their charter, they might happen to be invested. This University certainly has not done so. It had existed and flourished with all the privileges and powers which it enjoys at present, not only forty years, the usual period allotted for the prescription of patrimonial rights, but upwards of two hundred and forty years, before it either exercised, or found itself in a condition to exercise, its privilege of granting degrees, either in law or medicine; and there are Faculties besides that of surgery, in which it has never, as yet, thought of conferring a brevium or diploma: but, when the occasion shall serve, the power is ready. On the principle of prescription, however, it must follow, that, as almost all the degrees which the University has conferred, were granted after the period; when, by the ordinary rules of prescription, the University's right to grant such degrees must have ceased to exist: these degrees, and all degrees granted by other Universities, in similar circumstances, have been granted illegally, and are null and void.

The Memorialists might say, that the right of granting *brevia* in any particular Faculty, being included in their general right of granting *brevia, quavis licita Facultate,* they have, by exercising their privilege in any one case, secured their right to exercise it in all; but they need not insist on this, for the privilege in question is not a right to which, by the law of Scotland, the rules of prescription extend. It is a right, *meræ Facultatis,* one essential quality of all of which rights is, that they may be exercised, *quandocunque*: and a little reflection will show, that all Universities, and Corporations like Universities, must have rights or powers, which, from the nature of things, may lie dormant till needed, and be used or not used, according to circumstances and sound discretion; but, being rights *meræ Facultatis,* they can neither be lessened nor weakened by participation, nor lost by prescription.

In the meantime, the Memorialists humbly hope that they will be excused, if they state shortly some of the circumstances which lead them to believe that the present bill, so far as they are concerned, is not necessary; and that, if passed into a law, without some material alterations, it will, instead of doing good, do much harm.

In order to show that it is not necessary with respect to this University, the Memorialists have to state, that the establishments in the medical department are now nearly complete. It perhaps is not known to many who may be called on to vote on the bill, that for a century past, lectures have been given in the University of Glasgow during the winter session, viz. from the 1st of November to the 1st of May, on the theory and practice of medicine, and on anatomy and surgery, by Professors appointed by the crown. That for a long course of years, lectures have been given on chemistry, on materia medica, and pharmacy, and on midwifery, by Lecturers appointed and endowed by the University; and that some of those chairs were formerly filled by Cullen, and Black, and Robison, and Irvine.—That, of late years, Government has taken the rising state of the medical school in this University more immediately under its consideration, and three additional Professorships, viz. one on Natural History, another on Midwifery, and a third on Surgery, have been instituted and endowed by the crown.—That, above twenty years ago, this large and populous city, the second it is believed in point of numbers in the kingdom, was blessed in the establishment of an infirmary, on a scale proportioned to its population and the need of the country around; to the erection and maintenance of which, the University contributed liberally from their funds, and to the practice in which their medical students sedulously attend.—That, in the elegant and richly stored musæum, bequeathed to the University by the munificence of the late Dr. William Hunter, and in the collections accumulating under the hands of the different Professors, there is a rare and valuable collection of preparations, which are regularly exhibited and examined, in illustration of the lectures on anatomy, surgery, and midwifery.—That, a few years ago, the University received pecuniary aid, to a considerable amount, from Government; to which they added a large sum out of their funds, for the erection of an extensive range of Buildings, containing, among other accommodations, a chemical laboratory and lecture room, and a very complete anatomical theatre with all its necessary appendages, &c.: and that the number of medical students attending the University, thirty years ago, used to be from thirty to forty annually; but that, before the end of the war, they had increased to between three and four hundred. The Memorialists, therefore, are at a loss to see on what grounds they should be deprived of the right of granting diplomas in surgery; and they trust, that after what has been stated is impartially considered, the Legislature will not, without further inquiry, see it to be either just or necessary, or expedient, to degrade this ancient, and the Memorialists will say useful seminary of learning, and hold it up to the public as unworthy of being trusted with any hitherto unabused privilege of its charter.

It is understood by the Memorialists, that the bill in question has originated with the College of Surgeons in London; who, they learn, profess to act on the most liberal principles, and to be actuated by the desire of protecting the public against ignorant pretenders in surgery. The Memorialists have no wish to disagree with any other corporate body: but they think they have a right to ask, whether it be intended to be understood that this university is actuated by less honourable motives. With regard to zeal for the improvement of surgery, and the protection of the subject against ignorant pretenders, the Memorialists will not yield to the College of Surgeons of London, or to any College ; but they cannot help observing, and with regret, that they see nothing in the bill that promises to insure good and sufficient education ; and as to liberality, they remember, some years ago, another bill was brought into the House of Commons, the object of which bill was to place the whole power of granting diplomas in surgery in the hands of the College of Surgeons of London ; the illiberality of which was so obvious, and the grasping at the complete monopoly of surgical education so apparent, that it was rejected by that Honourable House. The present bill, indeed, seems to be more liberal ; for it proposes to share the right of granting diplomas between the three Colleges of Surgeons of London, Edinburgh, and Dublin. But to this university it will, in the hands of these three, be a monopoly still : for it requires little sagacity to foresee, that wherever the monopoly of the diploma goes, there the student must follow ; and, without imputing blame, or unworthy motives to any one, it is obvious that the students will very soon see it to be prudent, if they do not find it to be necessary, to attend such classes, and such only, as the three Colleges of Surgeons set up or patronize ; so that, in the course of a very few years, the old establishments will be supplanted, and in a great measure set aside ; and three new establishments will be set up, supported and guarded by a monopoly, against which no combination of talent and exertion will be able to contend.

As a proof that this is no chimera, it may be stated, that some years ago a law was passed (of which this university knew nothing till it felt the effects), whereby it was enacted, that no vessel, carrying fifty persons or upwards, shall be cleared out from any port of the United Kingdom, unless provided with a surgeon, having a certificate of having passed his examination before the College of Surgeons in London, Edinburgh, or Dublin. The consequence of which has been, that several students have left this university ; and others are convinced, that if the law remain in force, they must next year, however inconvenient it may be, go either to London, Edinburgh, or Dublin. The thing to be complained of in all this is, that this University, instead of meeting with the countenance and support of the Legislature as a public institution, is and will still farther be placed not on an equal footing with the three Colleges of Surgeons which the bill proposes to favour ; and lest it should be said or imagined that any partiality is expected, the Memorialists hereby declare, that they wish for nothing but that their charter be respected, which will place them, and do no more than place them, on an equality with these Colleges.

The Memorialists are sensible that they are not entitled to speak, on this business, for others ; at the same time, lest murmurings and complaints from other quarters follow, it is perhaps necessary to be known, and known in time, that the injurious and degrading consequences of the proposed law will not be confined to this University. They will speedily spread, and be felt severely by this large and populous town, and by the greater part of the west of Scotland, as well as by a considerable part of the north of Ireland, from which places most of the students at this university come. Every thinking man must be sensible, that the greatest favour which learning can receive, or Government can bestow, is to keep the expence of education moderate. If, however, the students of surgery throughout the empire must repair to one or other of the capitals of the three kingdoms, the expence will be more than the means of most of the students who come here will be able to bear.

The Memorialists only add, that they have nothing to ask. If their charter be left to them entire and confirmed, it is all they desire. And this may be effectually done, by adding the words, "and University of Glasgow," in that clause of the bill by which the right of granting diplomas is to be given to the Colleges; or it may be done, by adding a clause to the end of the bill, enacting, "That nothing in this bill shall be held as abrogating or impairing the rights and privileges conferred on the University of Glasgow by its foundation charters, which are hereby recognized and ratified and confirmed, any thing in this or any former bill to the contrary notwithstanding." But in whatever way Parliament shall see meet to dispose of this business, the Memorialists humbly hope and rest assured, that the honour, the credit, the interests, and the past services of this University, will not, as in the bill, be altogether overlooked or forgotten.

In the name and by appointment of the Senate of the University,

(Signed) Lockhart Muirhead, Vice Rector.

University of Glasgow, Feb. 24th, 1817.

---

For the information of our various readers, we insert the following Regulations relative to obtaining the Degree of M. D. in the University of Edinburgh.

Q. *F. F.* Q. *S. Statuta Solennia de Doctoratus in Medicina gradu in Academia Edinburgena Capessendo, a Facultate Medica Proposita, et in Posterum, Jubente Senatu Academico, Observanda.*

1. Nemo ad doctoratus in medicina gradum promoveatur, nisi Die Solenni, nempè primo mensis Augusti, vel die proximè sequente; nec prius quàm ipse annum ætatis suæ unum et vigesimum compleverit.

2. Nemo Candidatorum numero adscribatur priusquam triennium, saltem per sex menses quotannis, in hâc aut aliâ Academiâ, Medicinæ studio impenderit, et omnibus quas Scientia Medica complectitur Disciplinis, scilicet, Anatomiæ et Chirurgiæ; Chemiæ; Botanicæ; Materiæ Medicæ et Pharmaceuticæ; Medicinæ Theoreticæ; Medicinæ Practicæ; Prælectionibusque Clinicis, sub Medicinæ Professoribus habitis, operam dederit.

3. Quicunque honores Medicinæ ambierit, ante diem XXIV$^{um}$ Martii, consilium suum cum Facultatis Medicæ Decano communicet, et illi tradat Dissertationem Medicam Inauguralem, à seipso compositam, ut Professor aliquis, a Decano designandus, eam perlegat, si opus fuerit emendet, et perlectæ scriptam suam testificationem apponat. Cum dissertatione, tradat etiam Medicinæ Studiosus Decano Facultatis, Studiorum testimonium in hâc aut in aliâ Academiâ; atque Autographum his verbis: "Ego —— ——— gradum Doctoratûs in Medicinâ ambiens seriò et sanctè Medicinæ Professoribus et Almæ Academiæ Edinburgenæ assevero, et hoc scripto meo testatum cupio, me unum et vigesimum Ætatis Annum jam complevisse, (vel, si ita res se habuerit, ante diem solennem esse completurum), et esse liberum hominem, scilicet nullius Chirurgi, aut Pharmacopolæ, aut alius cujùsvis artificii Magistri servitio addictum, ut Discipulum, vel Tironem, vel Ministrum, qualis Anglicè dicitur *Apprentice.*"

4. Postea Quæstio à Facultate Medicâ, vel vivâ voce, vel scripto, privatim habenda est, de variis quas Scientia medica complectitur disciplinis; ut nemo nisi Literarum et Medicinæ scientiâ probè imbutus, Candidatorum numero adscribatur.

5. Die XXIV$^{to}$ mensis Junii, Candidatus, coram Facultate Medicâ, a duobus Professoribus interrogatus, progressum suum in Variis Disciplinis Medicis, suprà enumeratis, ulteriùs ostendat.

6. Candidato hactenus probato proponatur, ab aliquo Professorum, unus ex Aphorismis Hippocratis; et simul, ab alio Professore, Quæstio Medica quorum priorem à seipso explicatum et Commentario illustratum; posteriorem, unà cum Responsione idoneis argumentis confirmatâ, die IV$^{to}$ mensis Julii, Professoribus proponentibus Candidatus reddat; suumque demum Commentarium et Responsionem, die VI$^{to}$ mensis Julii, coram Facultate Medicâ defendat.

7. Si, his ritè peractis, Candidatus, promoveri merebitur, illi tradantur, Duæ Morborum Historiæ, cum Quæstionibus Subjunctis, ut, scripturâ, illas illustret, his commoda Responsa reddat; tum Historias ita illustratas, unà cum Responsis suis, die XIX$^{no}$ Julii Professoribus proponentibus tradat, atque eadem, die XXII$^{do}$ Julii coram Facultate Medicâ defendat.

8. Candidato, si, post primum periculum factum, probatus fuerit, Dissertationem suam inauguralem prelo subjicere liceat, cujus accuratè excusæ octo exemplaria, Facultatis Medicæ Decano, die XXII$^{do}$ Julii, tradat.

9. Si Candidatus, Dissertatione jam excusâ, tertiò a Medicinæ Facultate fuerit probatus, ejusdem Facultatis Decanus omnia quæ gesta fuerint Senatui Academico renunciabit; cujus approbatione et auctoritate Candidatus Dissertationem suam edere, eandemque in Comitiis Academicis, die anteà statuto, nempè I$^{mo}$ Augusti, defendere jubeatur: Tum, si Senatui placuerit, laboris tandem et studiorum præmium, summos in Medicinâ Honores, Gradum nempè Doctoralem, more solenni, consequatur.

10. Facultas Medica, quo major sit horum omnium solennitas, semper intra Academiæ Pomœria, Horâ Nonâ ante meridiem, diebus supradictis, conveniet. Et si quis candidatus, sine gravi causâ, horâ abfuerit statutâ, occasione neglectâ, ei, hâc vice, vel ad ulteriora pericula progredi, vel Gradum Doctoralem assequi, non licebit.

11. Exercitationes omnes anteadictæ, Linguâ Latinâ peragendæ sunt.

ALEX. MONRO, jun.
Prof. Anat. & Chir. Facult. Med. Dec.

E Tabulis Academiæ, jubente Senatu Academico, describenda curavit,
*ANDREAS DUNCAN, jun. Med. Leg. Pr. Reg. Acad. à Secretis et Bibliothecarius.*

*Data Edinburgi, in Acad. Jac. Reg.*
*Anno Salutis Humanæ* M.DCCC.XIV.

---

### MEDICINA LAICA.

1. *A Cadavery at Mazara in Sicily.* "In one of the lesser churches Mr. Galt had an opportunity of seeing a Cadavery. A large well-lighted room, containing about a hundred dead men, women, and children, placed without any veil or mask to hide the horrible look with which they seemed to regard the living. Some of them are in niches; others are lying on the floor. Some are yet entire; others are half mouldered away. The mode of preparing the dead for this hideous and disgusting exhibition is very simple. The body cleansed from the bowels, is placed in a vault, from which the air is carefully shut out. In the course of three months the whole moisture is exuded, and the corpse having become quite dry, is then removed into the cadavery.

2. *Picture of a Leper in Sicily.* "One of them was reduced, says Mr. Galt, to the most frightful spectacle in which the human form can be retained. His skin was shrunk and black; his neck and limbs swollen, and covered with a disgusting crust; and his teeth, long and yellow, seemed to be only sticking in a mass of putrefaction. Yet he could articulate; but his tones were, if possible, more horrible than his figure.

3. *Turkish Mode of Sepulture at Salonika.* "In the course of walking round this city, we had occasion to pass through one of the cemeteries; but the horrible effluvia from the graves obliged us to alter our course. The Turks do not make use of coffins. Having deposited the dead, they place over the body a few thin pieces of wood, and then cover it with earth. Heavy rain has often the effect of opening passages down to the putrifying mass, occassioning that pernicious and terrible smell which we experienced, and to which may, in some degree, be attributed the frequency of pestilential diseases in Turkey." *ib.*

4. *A Turkish Bedlam at Constantinople.* "The building on the outside is plain and simple; but the court, around which the cells are constructed, is built of marble, and the arcades resemble those of the Royal Exchange of London. Several of the patients, almost entirely naked, were fastened by chains fixed to iron collars round their necks, and sat at the grating of their windows, like savage animals in cages. The physician of this hospital was old and venerable. He told us that there were four great classes of insanity, distinguished by their causes.—1st. Madness, which came from fevers. 2d. Melancholy, which came from fires in the city, or other great misfortunes. 3d. Phantasy, which came from wrong conceptions of the imagination. 4th. Fits of delirium, which were produced by the magical devices of enemies. The first kind of insanity, he assured us, was rarely cured; but the second and third, often and easily. The fourth was incurable, unless the enchanter could be discovered, and obliged to break up his spell!"

5. *The Plague.* "When the great population of Constantinople is considered, the narrowness of the streets, the quantity of putrid matter constantly lying in them, and the covered bazars excluding the fresh air, it is not surprising, in a climate subject, occasionally, to extreme heats, that the inhabitants should often be visited by pestilence. The nature of the plague, as far as I am able to judge, is still very imperfectly known in Turkey. The terror which the very name inspires is a sufficient proof that few have had the courage to examine it attentively. The infected are shunned with abhorrence, and the dread of the contagion seems to preclude the hope of its effects being properly investigated. The substance of the information that I collected from a person that had endured the disease, and attended the infected for some time in one of the hospitals, is as follows:

"The symptom first perceived by the patient is a painful sensation, resembling the pricking of a lancet, or the sting of an insect. The sensation is so sharp, that if it takes place in sleep, it never fails to awaken the person. Soon after, an obtuse pain is felt in the head; a fever ensues; and in the course of twenty-four hours, tumours make their appearance in the groins and arm-pits. If the disease is to prove fatal, the patient never again falls asleep; but the fever and tumours increase till he dies: otherwise, the head-ache and fever abate at the end of the twenty-four hours, and he enjoys repose. Death generally takes place before the suppuration of the tumours: when the suppuration has arrived at maturity, death is not apprehended; but it is a mistake to suppose that the patient, after recovery, is not again liable to the disease.

"There are several kinds of the plague, and only one of them secures the patient from subsequent attacks. Perhaps it would be more correct, to consider this kind as the full development of the distemper. It is called the King of the Pest, from being attended with a remarkable eruption on the spot where the patient first felt the infection: this name is derived from the eruption, to which, indeed, it is particularly applied. The King makes its appearance at the same time as the tumours, and grows to maturity with them. After suppuration, it protrudes a quantity of corrupted flesh, which is cut off, and which the Greeks preserve, dividing it among their friends, who believe the wearing of it will secure them from infection. There is no instance of any one dying with the King of the Pest, or of being a second time infected. If ever the disease is to be prevented by inoculation, perhaps it is from the matter of this eruption.

"The great prevention of the contagion is, the interruption of intercourse; but there is a species of vinegar, which, when drawn up into the nostrils, is supposed to afford no small degree of security. It is called the Vinegar of the Four Thieves, having been invented by four wretches of Marseilles, who, during the great plague there, entered and plundered the infected houses with impunity. This fact seems to be universally admitted, that strong odours are of great utility in the prevention of the disease; the obvious inference from which is, that proper fumigations would reduce its violence. Fruits and humid substances do not retain or communicate the infection; but all dry substances, and living animals, convey it; and the latter are liable, themselves, to the disease, the symptoms and progress of which are similar to those which take place on the human subject. In the course of the malady, the patient must carefully abstain from gross food of every kind, and also from crude fruits, living, sparingly, on the most meagre diet."—*Galt's Travels in the Levant.*

---

*Epilepsy cured by the Operation of the Trephine.* (Extract of a Letter from a Physician to Dr. Johnson.) In looking over my note-book, I find a memorandum to the following purport, made in the year 1807. Imperfect as the statement is, you may, perhaps, not think it entirely unworthy of being placed upon record. Although the case did not occur under my own eye, I, from my knowledge of the narrator, feel no hesitation in vouching for its accuracy: "An experienced and highly respectable surgeon (in one of the midland counties) informs me that, some years since, he was consulted respecting a lad who, from an early age, had been subject to epileptic fits. All the usual remedies were tried without effect. At length, the boy, from one of those accidents so frequently occurring in coal-pits, got his skull severely fractured. The narrator was called to him, aud found it necessary to employ the trephine in order to elevate a depressed portion of the parietal bone, and remove some extraneous substances lodged between it and the dura mater. Convalescence was marked by no peculiar or untoward circumstance; nor did the epilepsy recur so long as the surgeon continued his attendance. Many months had elapsed ere he again saw the lad; who was so uncommonly improved in his general appearance that the surgeon did not, at first, recognize him. Upon inquiry, he declared that the fracture of the skull had proved a most fortunate occurrence: for, since that time, he had uniformly enjoyed excellent health, and had experienced no return of the epileptic fits."

I have somewhere seen an account of a proposal made by a French surgeon to employ the trephine to the cranium, as a last resource in cases of otherwise incurable epilepsy. Who is this surgeon? What is the principle upon which removal of a small portion of the calvaria operates in the cure of epilepsy? What are the peculiar circumstances under which such an operation is justifiable? What the precise situation in which it may be most advantageously performed? For information on this subject, I shall feel obliged to any of your readers whose knowledge and experience may enable them to answer all or part of these questions, in a satisfactory manner. Any surgeon, who may have met with a similar case, will highly oblige me by its communication; as I am anxious to accumulate facts capable of throwing light on this curious and interesting inquiry.

---

A Letter has very lately been received by Dr. Baillie from Dr. Crichton at Petersburgh, giving an account of the successful employment of *Pix liquida* in pulmonary consumption. Dr. C. had made trial of it in three cases, one of which was cured, and the other two much relieved by the use of it. The pitch, placed over a charcoal fire, is slowly evaporated, so as to fill the apartment with its fumes, which are consequently inhaled by the patient. As it is desirable that the actual sanative powers of any substance thus offered to the medical world should be as promptly as possible ascertained, and as there is no class of disorders in which it is more justifiable to resort to an *anceps remedium*, we anticipate many trials of the *new* remedy, and we shall feel obliged to any of our Correspondents for an early account of the results of the experiments they may institute on this subject.

---

Professor Brande, in a Lecture lately delivered at the Royal Institution, pointed out the difficulty of detecting arsenic by the common tests usually employed for that purpose; and shewed, that the yellow precipitate which white arsenic produces in solution of nitrate of silver, exactly resembled that which phosphoric acid occasions, and that both are soluble in ammonia. He also exhibited the effect of the juice of onions upon a solution of sulphate of copper, which caused a green tint, not to be distinguished from the colour produced by small quantities of arsenic. Mr. Brande observed, therefore, that in any case of importance, no reliance should be placed in the above tests; which, in the hands of ignorant persons, would inevitably lead to error, and that the *separation of the metal itself could only be relied upon.* He was led to these remarks in consequence of a recent trial in Cornwall, and the subject is of the highest importance, as connected with judicial evidence, in cases of supposed suicide.

Messrs Majendie and Pelletier have communicated to the Academy of Sciences at Paris, an interesting discovery upon Ipecacuanha. It appears that they have succeeded in separating the principal substance, to which the good effects of ipecacuanha in medicine are owing, from those adjuncts which give it that odour and smell so disagreeable to invalids. They have named this principal substance *hemetine*. A great number of experiments and observations have been made, which fully confirm the truth of the discovery. *Phil. Mag.*

*Medical Benefit Society.*—At the first Annual General Court the following gentlemen, Dr. Luke, Dr. Drever, Joseph Hayes, Esq. A. T. Thomson, Esq. R. S. Wells, Esq. and Reginald Williams, Esq. went out of the Direction by rotation ; and Dr. G. G. Currey, Dr. Outram, H. L. Thomas, Esq. R. R. Pennington, Esq. Neville Wells, Esq. and Edward Browne, Esq. were elected.—The First Anniversary of this admirable Institution was commemorated at the Freemasons' Tavern, on Saturday May the 10th, when between forty and fifty gentlemen, among whom were some of the most distinguished members of the profession, assembled. The President of the Royal College of Physicians, Dr. Latham, to whose philanthropy the Society owes its foundation, was in the Chair.—Persons desirous of information respecting the nature of this Society, will be informed of every particular, by applying to Mr. Best, Secretary, No. 15, Tavistock Street, Covent Garden.

Mr. Thomson will commence his Summer Course of Lectures on General and Medical Botany, on Tuesday the 3d of June, at two o'clock, in the Anatomical Theatre, Blenheim Street.

